# Selenium Supplementation during Puberty and Young Adulthood Mitigates Obesity-Induced Metabolic, Cellular and Epigenetic Alterations in Male Rat Physiology

**DOI:** 10.3390/antiox11050895

**Published:** 2022-04-30

**Authors:** Gabriela de Freitas Laiber Pascoal, Gabriela Machado Novaes, Monique de Paula Sobrinho, André Bubna Hirayama, Inar Alves Castro, Thomas Prates Ong

**Affiliations:** 1 School of Pharmaceutical Sciences, São Paulo 05508-000, Brazil; laiber@usp.br (G.d.F.L.P.); gabriela.mnovaes@usp.br (G.M.N.); moniquepaula@alumni.usp.br (M.d.P.S.); inar@usp.br (I.A.C.); 2 Medical School, University of São Paulo, São Paulo 01246-000, Brazil; a.hirayama@hc.fm.usp.br; 3 Food Research Center (FoRC), São Paulo 05508-000, Brazil

**Keywords:** selenium, obesity, male physiology, epigenetics reprogramming

## Abstract

Selenium (Se) role in obesity is not clear. In addition, information on Se’s role in male physiology, specifically in obesity, is scarce. We conducted this study to evaluate the efficacy of Se supplementation, specifically during puberty until young adulthood, against obesity-induced deregulation of metabolic, cellular, and epigenetic parameters in epididymal fat and/or sperm cells in a rat model. High-fat-diet consumption by male rats during puberty and young adulthood significantly increased body weight, adipocyte size, oxidative stress, deregulated expression of genes associated with inflammation (Adiponectin, IL-6, TNF-α), adipogenesis (CEBPα), estrogen biosynthesis (CYP19) and epigenetic processes in epididymal adipose tissue (Dnmt3a), as well as altered microRNA expression vital for spermatogenesis in sperm cells (miR-15b and miR-497). On the other hand, Se supplementation significantly decreased oxidative stress and mitigated these molecular/epigenetic alterations in epididymal adipose tissue or sperm cells. Our results indicate that selenium supplementation during puberty/young adulthood could improve male physiology in the context of obesity. In addition, it suggests that Se could potentially positively affect offspring health.

## 1. Introduction

Obesity is among the most alarming global public health problems [1]. It is associated with premature mortality and increases the burden of non-communicable diseases [2]. Importantly, in men, obesity is also linked to deregulation of reproductive physiology [3]. Mechanisms whereby obesogenic conditions lead to poor sperm quality and male infertility include hyperestrogenism, elevated testicular and sperm levels of inflammatory mediators and reactive oxygen species (ROS), deregulation of spermatogenesis, and sperm epigenetic perturbation [4].

Selenium (Se) is an essential micronutrient with several functions in humans, such as thyroid regulation, anti-inflammatory actions, and antioxidant activities [5,6]. These functions are exerted through several selenoproteins in which Se is incorporated as the amino acid selenocysteine [6]. Among them, Glutathione peroxidases (GPXs) encompass a key family of antioxidant enzymes involved in the removal of hydrogen peroxide, lipid hydroperoxides, and phospholipid and cholesterol hydroperoxides [6]. Importantly, GPX activity is dependent on Se’s nutritional status [5]. The involvement of Se in metabolic diseases is a matter of increased attention in the literature [7], although its precise role in obesity is still not clear [8]. A clinical study showed an association between Se nutritional status and metabolic risk factors in men with visceral obesity [7].

Se also presents a key biological role in male reproductive physiology [2]. It is a constituent of selenoproteins that protect spermatozoa against ROS and simultaneously increase motility and sperm viability [9]. Se deficiency during spermatogenesis results in fertility disorders and abnormal semen parameters [9]. Spermatogenesis comprises intense epigenetic remodeling at the level of DNA methylation, histone modifications, and microRNA levels [10,11,12]. Adequate sperm epigenome patterns are fundamental for fertilization and proper embryo/fetal development [13,14]. While some studies have shown the effects of Se on these epigenetic marks in cancer cells [15,16], its role in epigenetic processes during obesity and/or spermatogenesis is unknown.

Se deficiency is frequently observed in infertile men in different populations [17]. It has been proposed that selenium nutritional status could reflect the fertility competence of the young population, and its monitoring would represent a strategy orienting dietary adjustments to attain normal reproductive function [17]. In addition, some studies showed a direct correlation between obesity and plasma selenium deficiency [18,19]. However, although Se’s actions are closely associated with carbohydrate and lipid metabolism, its potential roles in obesity development and in adipocyte metabolism are not clear [20]. Furthermore, information on Se’s role in male physiology, specifically in obesity, is scarce [18]. Currently, obesity is a prevalent condition in male adolescents [21]. In addition, school children aged 8–13 years with excess weight were shown to have a poor selenium status, a condition that could contribute to low antioxidant protection [22]. Because puberty is a key window of susceptibility to spermatogenesis deregulation induced by obesity and/or Se deficiency, supplementing obese male adolescents with Se could represent a potential clinical strategy to improve their sperm epigenetic pattern. Because epididymal fat impacts epididymis physiology and consequently sperm epigenetic maturation at the level of microRNAs [23,24,25], Se supplementation in this context could also ameliorate the function of that fatty tissue with beneficial effects on male physiology. This could have an impact not only on men themselves but potentially on their future descendants. Importantly, from a female perspective, experimental evidence already highlights possible Se dietary supplementation treatment for gestating and lactating mothers to promote their metabolic health and prevent intrauterine growth retardation, which could affect their progeny’s future health in adulthood [26].

Thus, we conducted this study to evaluate the efficacy of Se supplementation, specifically during puberty until young adulthood, against obesity-induced deregulation of metabolic, cellular, and epigenetic parameters in epididymal fat and/or sperm cells in a rat model.

## 2. Materials and Methods

### 2.1. Animal Model

This study was approved by the Ethics Committee on the Use of Animals of the School of Pharmaceutical Sciences of the University of São Paulo (CEUA/FCF/USP, n° 571). Male Sprague Dawley rats, aged 3 weeks, were maintained in a temperature- and humidity-controlled animal facility under a 12-hour light-dark cycle (6:00 am–6:00 pm). The animals were kept in polypropylene cages (*n* = 4/cage) with stainless steel lids and containing previously sterilized wood shavings, changed every other day. Forty-five male Sprague Dawley rats at 4 weeks of age were randomly assigned to 3 groups with 15 rats in each group. For a period of 9 weeks (from the 4th week to the 13th week of age), control group (CO) received a control diet (AIN-93G [27]; 0.15 ppm Se, as sodium selenate); obese group (OB) received a high-fat diet based on lard (0.15 ppm Se, as sodium selenate), with 60% of calories coming mainly from lipids; and obese group supplemented with Se (OBSe) received the same high-fat diet (0.15 ppm Se, as sodium selenate) together with drinking water containing sodium selenate (0.45 ppm Se, Merck, Darmstadt, Germany). Diets were purchased from Prag Soluções (Jaú-S, Brazil). Diet composition is provided in Supplementary Table S1. Animals’ weights, diet, and water consumption were recorded on alternate days. At 13 weeks of age, all male rats were subjected to 3–4% of inhalatory isofluorane. Euthanasia was performed by cardiac puncture (exsanguination), and the blood was stored at −80 °C until the beginning of the analyses. After this procedure, epididymal adipose tissue and liver samples were placed in liquid nitrogen and stored at −80 °C for further metabolic, cellular, and epigenetic analyses.

### 2.2. Histopathology of Epididymal Adiposetissue and Testicles

For the morphometrical analysis, the epididymal adipose tissue and testicles were collected and fixed in 10% buffered formaldehyde and paraffin. Sections of 5.0 μm were used for histological H&E slides. Testicles analysis was performed according to Johnsen score scale 1 ± 10 [28]. Mean value score for 100 seminiferous tubules was calculated for every testis in each section. For histological analysis, slides were obtained in an image capture system consisting of a trinocular microscope (Axioskop 2, Zeiss, Oberkochen, Germany) and a digital camera (Axiocam, Zeiss, Germany) [28]. The samples were analyzed by a pathologist.

### 2.3. Plasma Cholesterol and Fractions

This analysis was based on the classical enzymatic colorimetric method and performed by the AFIP Medicina Diagnóstica laboratory (São Paulo, Brazil).

### 2.4. Oxidative Stress–Malondialdehyde Levels (MDA)

This analysis was performed on epididymal adipose and liver tissue. The concentration of MDA was determined by reversed-phase high-performance liquid chromatography (HPLC) [29]. The sample emulsion was submitted to alkaline hydrolysis and was incubated and centrifuged, and the extraction of MDA by *n*-butanol was analyzed in an isocratic condition [29]. Samples were analyzed by Synergy HT Spectrophotometer (BioTek, Winooski, VT, USA) using Gen5 software (BioTek).

### 2.5. Activity of Antioxidant Enzymes

These analyses were performed on liver tissue based on the classical spectrophotometrical enzyme assays Catalase (CAT) activity [30], Superoxide dismutase (SOD) activity [31], and Glutathione peroxidase (GPx) activity [32].

### 2.6. Analysis of Epididymal Adipose Tissue Expression of Genes Associated with Inflammation, Adipogenesis, Estrogen Biosynthesis, and Epigenetic Processes

Primers were custom designed using the OligoAnalyzer^TM^Tool (IDT, São Paulo, Brazil). The expression of the following genes: PPARγ, CEBPa, CEBPb, Adiponectin, CYP19, IL-6, TNF-a, DNMT3a, and DNMT1 were estimated in epididymal adipose tissue of animals from all groups. Around 100 mg of liquid nitrogen-sprayed in epididymal adipose tissue was homogenized in TRIZOL reagent for total RNA extraction, as described by Chomzynski and Sacchi [33]. One microliter of the solution was placed in a Nano Drop 2000 apparatus (Thermo Scientic, São Paulo, Brazil) for RNA quantification. When samples presented a ratio 260/280 nm over 2, cDNA was synthesized with reverse transcriptase from 1 µg of RNA. QuantStudio 7 Flex™ Real-Time PCR System (Life Technologies, Waltham, MA, USA) was used to determine gene expression profile as described [34], using SYBER Green reagent (Invitrogen, Life Technologies) as the fluorescent marker. Primer’s details are provided in Supplementary Table S2. Β-actin gene expression was used as control.

### 2.7. Histones Modifications

H4k16ac and H3k4me3 marks were evaluated by immunohistochemistry [35]. Epididymal adipose tissue was collected and fixed in H&E slides in sections of 5.0 μm. The dilutions of histones H4k16ac and H3k4me3 antibodies were 1/100 and 1/200, respectively [36].

### 2.8. Analysis of Sperm MicroRNA Levels

The caudal epididymis and ductus deferens were punched and moved to a culture plate containing M2 medium (M2 medium with HEPES, without penicillin and streptomycin, stereo filter, appropriate for the mouse embryo; Sigma-Aldrich, St. Louis, MO, USA), where it was incubated for 1 h at 37 °C. Samples were washed with PBS and incubated with somatic cell lysis buffer (SCLB; 0.1 SDS, 0.5% Triton X-100 in diethylpyrocarbonate water) for 1 h, according to Platts et al. (2007) [37]. SCLB was washed with two baths of PBS, and the purified sperm sample (minimum 95% purity as assessed by microscope) was pelleted and used for miRNA analysis. For total miRNA extraction, the mirVanaTM miRNA Isolation Kit (ThermoFisher, Waltham, MA, USA) was used. Then, the reverse transcriptase reaction was performed with a specific primer for miRNAs (hsa-miR-200c, hsa-miR-497, hsa-miR-15b; see Supplementary Table S3). The qPCR technique was performed using the TaqMan technology (Applied Biosystems^®^ TaqMan MicroRNA Assays kits, Waltham, MA, USA) according to the manufacturer’s instructions. For endogenous control, miRNA RNU 49 was used. The analysis was performed with an Applied Biosystems^®^ 7500 Real-Time PCR thermocycler, and quantification was performed by calculating ^ΔΔ^Ct.

### 2.9. Statistical Analysis

Statistical analysis was conducted with GraphPad Prism 9.0 (GraphPad software Inc., San Diego, CA, USA). All data were tested for normality. One-way ANOVA was used, followed by Tukey’s multiple comparisons tests. Specifically, for epididymal adipocyte and testicular morphological statistical analysis, chi-square test and Student’s *t*-test were used. *p* ≤ 0.05 was accepted as threshold of statistical significance. Data are presented as mean and standard error of the mean (SEM).

## 3. Results

### 3.1. Body Weight and Daily Intake

No differences were observed between CO and OB groups regarding initial weight (*p* > 0.05), daily feed intake (*p* > 0.05), and daily water intake (*p* > 0.05) parameters (Supplementary Figure S1). Regarding Se daily intake, this was as follows: the CO group (7.2 ± 2.4 µg/day/animal); the OB group (6.5 ± 2.5 µg/day/animal) and the OBSe group (20.8 ± 7.6 µg/day/animal). The OBSe group ingested 3.2× Se levels (*p* = 0.0006) compared to OB group. Compared to the CO group, the OB group presented a higher (*p* = 0.0089) final weight. No differences were observed between OB and OBSe groups regarding this parameter (*p* > 0.05) (Supplementary Figure S1).

### 3.2. Histopathology of Epididymal Adipose Tissue and Testicles

**Figure 1:**
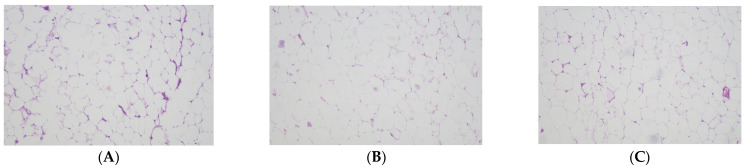
Representative photomicrographs of epididymal adipose tissue stained with hematoxylin-eosin. (**A**) CO group; (**B**) OB; (**C**) OBSe. All images are shown at ×100 magnification. Adipocyte sizes were larger (*p* = 0.0072) in OB group compared to CO group, while no differences (*p* > 0.05) were observed between OB and OBSe groups, according to chi-square test and Student’s *t*-test.

**Figure 2:**
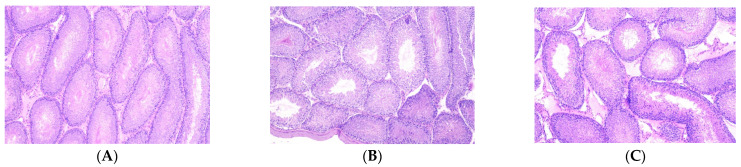
Testicular architecture. Representative photomicrograph of testicular tubules stained with hematoxylin-eosin. (**A**) CO group; (**B**) OB; (**C**) OBSe. All images are shown at ×10 magnification. No statistically significant difference (*p* > 0.05) among groups according to chi-square test and Student’s *t*-test.

Compared to CO group, OB group had larger (*p* = 0.0072) adipocyte size while OBSe group showed no difference (*p* > 0.05) regarding this parameter. No differences (*p* > 0.05) were observed between OB and OBSe groups regarding adipocyte size (Figure 1). No differences (*p* > 0.05) were observed among all groups regarding testicular architecture (Figure 2).

### 3.3. Total Cholesterol and Fractions

**Figure 3:**
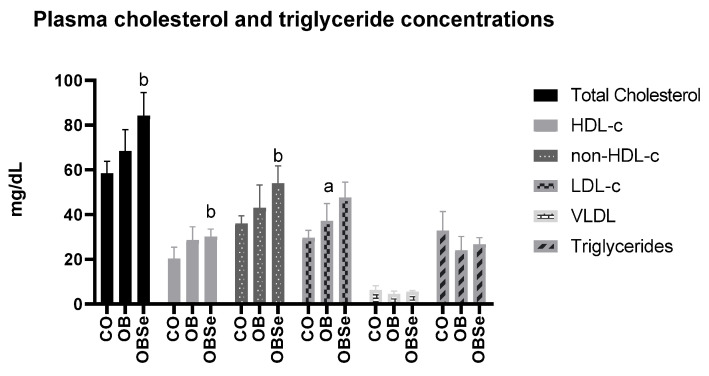
Effects of high-fat obesogenic diet and Se supplementation on total cholesterol and fractions and triglycerides plasmatic levels. Data represent the mean ± SEM (*n* = 5/group). Statistically significant difference (*p* ≤ 0.05) compared to ^a^ CO or ^b^ OB group according to one-way ANOVA followed by Tukey’s multiple comparisons test.

No differences were observed between OB and CO groups regarding total (*p* > 0.05), HDL (*p* > 0.05), and non-HDL plasma cholesterol levels (*p* > 0.05) (Figure 3). Compared OB group, OBSe group presented higher total (*p* = 0.0032), HDL (*p* = 0.0067) and non-HDL plasma cholesterol levels (*p* = 0.0217). Compared to CO group, OB group presented higher (*p* = 0.0017) LDL cholesterol levels, while there was no difference (*p* > 0.05), between OB and OBSe groups regarding this parameter. There was no difference between CO and OB groups regarding very-low-density lipoprotein (VLDL) (*p* > 0.05) and triglycerides levels (*p* > 0.05) (Figure 3). In addition, there was no difference between CO and OB groups regarding VLDL (*p* > 0.05) and triglycerides levels (*p* = 0.8302).

### 3.4. Oxidative Stress–MDA Levels

**Figure 4:**
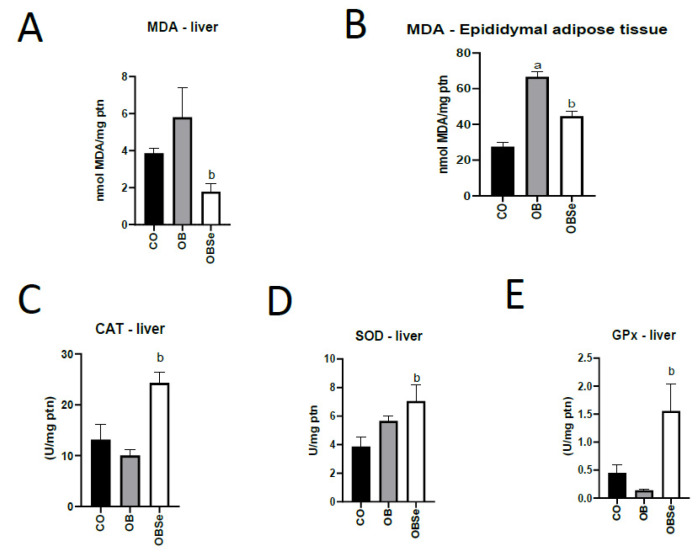
Effect of high-fat obesogenic diet and Se supplementation on MDA levels in liver (**A**) and epididymal adipose (**B**) and hepatic antioxidant enzyme activities (CAT (**C**), SOD (**D**) and GPx (**E**)). Data represent the mean ± SEM (*n* = 5/group). Statistically significant difference (*p* ≤ 0.05) compared to ^a^ CO and ^b^ OB groups according to one-way ANOVA, followed by Tukey’s multiple comparisons test.

No differences (*p* > 0.05) were observed between CO and OB groups regarding hepatic MDA levels (Figure 4). Compared to the OB group, the OBSe group presented lower (*p* = 0.0392) hepatic MDA levels. Compared to the CO group, the OB group presented higher (*p* < 0.0001) epididymal adipose tissue MDA levels. Compared to the OB group, the OBSe group presented lower (*p* = 0.0006) epididymal adipose tissue MDA levels (Figure 4).

### 3.5. Antioxidant Activity

No differences (*p* > 0.05) were observed between CO and OB groups regarding CAT, SOD and GPx activity (Figure 4). Compared to OB group, OBSe group presented higher antioxidant activity of CAT (*p* = 0.0002), SOD (*p* = 0.0310) and GPx (*p* < 0.0001) enzymes (Figure 4).

### 3.6. Gene Expression

**Figure 5:**
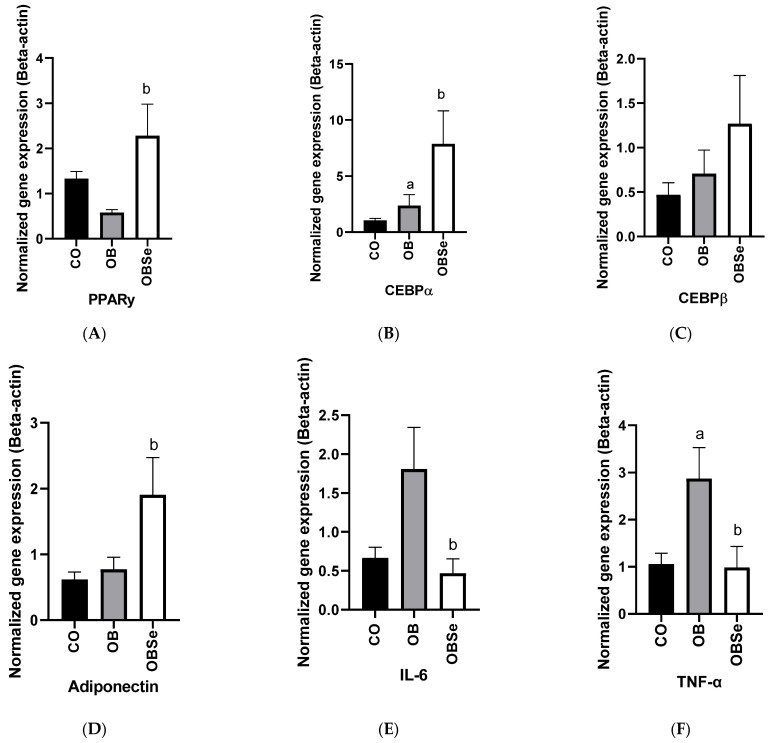

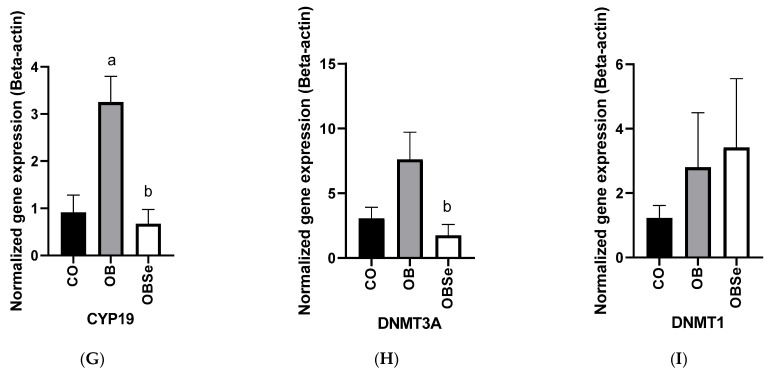
Effect of high-fat obesogenic diet and Se supplementation on gene expression in epididymal adipose tissue: (**A**) PPARy; (**B**) CEBPα; (**C**) CEBPβ; (**D**) Adiponectin; (**E**) IL-6; (**F**) TNFα; (**G**) CYP-19; (**H**) DNMT3A; (**I**) DNMT1. Data represent the mean ± SEM (*n* = 5/group). Statistically significant difference (*p* ≤ 0.05) compared to ^a^ CO and ^b^ OB groups according to one-way ANOVA, followed by Tukey’s multiple comparisons test.

Compared to the CO group, no differences (*p* > 0.05) were observed between the OB group regarding the expression of PPARy. Compared to the OB group, OBSe groups presented a higher (*p* = 0.0268) expression of PPARγ. Compared to the CO group, the OB group presented a higher (*p* = 0.0317) expression of a CEBPα gene. Compared to the OB group, the OBSe group presented a higher (*p* = 0.0098) expression of the CEBPα gene. No differences (*p* > 0.05) were observed between CO and OB groups and between OB and OBSe groups regarding the expression of the CEBPβ gene. No differences (*p* > 0.05) were observed between CO and OB groups regarding the expression of the adiponectin gene. Compared to the OB group, the OBSe group presented a higher (*p* = 0.0498) expression of the adiponectin gene. No differences (*p* > 0.05) were observed between CO and OB groups regarding the expression of IL-6 and DNMT3A. Compared to OB, the OBSe group presented lower expression of IL-6 (*p* = 0.0467) and DNMT3A genes (*p* = 0.0288). Compared to CO group, OB group presented higher expression of TNFα (*p* = 0.0382) and CYP19 (*p* = 0.0033). Compared OB, OBSe presented lower expression of TNFα (*p* = 0.0407) and CYP19 (*p* = 0.0015). No differences were observed between CO and OB group (*p* > 0.05) and between the OB and OBSe group (*p* > 0.05) regarding the expression of the DNMT1 gene. See Figure 5.

### 3.7. Histones Modifications

There was no statistical difference between the CO and OB group (*p* > 0.05) and between OB and OBSe (*p* > 0.05) regarding the expression of H4k16ac and H3k4me3 (Supplementary Figure S2).

### 3.8. Sperm MicroRNA Expression

**Figure 6:**
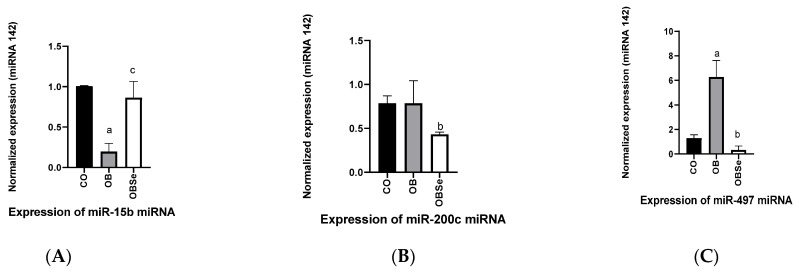
Effect of high-fat obesogenic diet and Se supplementation on sperm miRNA levels: (**A**) miR-15b; (**B**) miR-200c; (**C**) miR-497. Data represent the mean ± SEM (*n* = 7/group). Statistically significant difference (*p* ≤ 0.05) compared to ^a^ CO and ^b^ OB groups according to one-way ANOVA, followed by Tukey’s multiple comparisons test. Tendency of Statistically significant difference (*p* ≤ 0.07) compared to ^c^ CO according to one-way ANOVA, followed by Tukey’s multiple comparisons test.

Compared to the CO group, the OB group presented a lower (*p* = 0.0441) expression of miRNA has-miR-15b (Figure 6). Compared to the OB group, the OBSe group presented a tendency of increased (*p* = 0.0727) expression of this miRNA. No differences (*p* > 0.05) were observed between OB and CO groups regarding the expression of miRNA has-miR-200c. Compared to the OB group, the OBSe group presented a lower (*p* = 0.0357) expression of miRNA has-miR-200c (Figure 6). Compared to the CO group, the OB group presented a higher (*p* = 0.0025) expression of miRNA has-miR-497. Compared to the OB group, the OBSe group presented a lower (*p* = 0.0003) expression of miRNA has-miR-497 (Figure 6).

## 4. Discussion

We observed that Se supplementation did not alter final body weight and food intake. This suggests that at the level of intake in our study Se was not toxic. Aspects such as selenium dose and chemical form should be taken into consideration in the context of obesity as they can influence outcomes and even promote weight gain [38]. Se (0.45 ppm diet) exerted anti-obesogenic effects at the level of weight gain in adult male mice treated with a high-fat diet [39]. Of notice, a recent clinical study showed that Se supplementation (240 µg selenomethionine/day) for three months reduced the body weight of obese/overweight adult individuals. Thus, these experimental and human studies reinforce Se as a potential protective micronutrient in the context of obesity treatment.

Se effects as a hypocholesterolemic agent are still equivocal and depend on dosage and individual cholesterol baseline levels [40]. While in some in vivo studies, Se supplementation did not affect total plasma cholesterol in mice [41], we observed that Se increased plasma HDL-c levels. In addition, in our study, Se did not affect plasma LDL-c, VLDL, and triglycerides levels. These results are in accordance with a recent meta-analysis, where authors concluded that selenium supplementation did not affect triglycerides and VLDL-LDL-cholesterol levels [42].

Among obesity’s diverse deleterious actions, the deregulation of male reproductive physiology, affecting sperm quality and fertility, merits attention [4]. This has been associated with obesity-induced ROS production, particularly in the region close to the epididymis, where the last stage of sperm maturation occurs [18]. Here we showed that high fat diet-induced obesity led to increased oxidative stress in rat epididymal adipose tissue. Importantly, Se supplementation inhibited oxidative stress in epididymal adipose tissue. Because of the close physical and biochemical connection between this adipose tissue and the epididymis [18,43], Se protective effects may have also occurred in the latter tissue. This is important since the timing of the transit of sperm through the epididymis represents the developmental window where sperm are the most susceptible to oxidative damage [18]. In addition, we observed that high-fat diet-treated animals presented increased gene expression of proinflammatory cytokines TNF-α and interleukin 6 in epididymal adipose tissue. On the other hand, Se presented anti-inflammatory actions in this adipose tissue by inhibiting the expression of these genes and inducing the expression of adiponectin, an anti-inflammatory cytokine. Similarly, selenate (0.5 mg/kg b.w) presented in obese mice these oxidative stress and inflammation inhibitory effects in serum and total adipose tissue [38]. Of notice, results from our study were observed at a level of selenate intake 10 times lower. McPherson et al. (2019) showed that male obese mice supplementation for 12 days, during the critical epididymal window, with the mix of antioxidants containing Se restored oxidative stress in sperm cells [18]. Thus, our study expands the knowledge on Se potential in this context by showing its antioxidant and anti-inflammatory protective effects, specifically on male physiology in obesity.

Se antiobesity effects could be related to modulation of adipogenesis [20]. Se participates in key pathways involved in adipocyte differentiation and metabolism [44]. PPARγ is a key transcription factor for adipocyte function that presents decreased expression and activity in obesity [45]. In addition, Se supplementation could increase PPARγ expression in these animals, an effect that could be related to its anti-inflammatory actions, as proposed before [46]. On the other hand, in a similar mice study [47], Se antiobesity actions involved opposite effects on PPARγ. It has been highlighted that Se effects on this transcription factor would be influenced by Se characteristics and study design [20]. In addition, these authors [47] found that selenate-administration inhibition of bodyweight gain was largely due to a decrease in adipose tissue mass, which can be attributed at least in part to decreased adipocyte hyperplasia and altered adipogenesis (including CEBPα increased expression) and lipid metabolism in adipocytes. However, in our study, Se did not alter obesity-induced adipocyte size. Thus altogether, in our study effects of Se on PPARγ and CEBPα expression could indicate a restoration of epididymal fat adipocyte metabolic function during obesity.

Literature data report that obesity can increase DNMT expression and activity [48,49] and that increased expression of Dnmt3a in the adipose tissue may contribute to obesity-related inflammation [50]. Our study showed increased expression of the DNMT3a gene in epididymal adipose tissue rats treated with a high-fat diet during puberty and young adulthood. Similarly, obese children and adolescents presented higher plasma DNMT3A expression [48]. On the other hand, as previously reported in obese patients [49], we did not observe changes in the expression of the DNMT1 in obese animals. Information on Se epigenetic modulatory potential in metabolic disease context is limited [51]. We are unaware of such Se epigenetic studies in the context of obesity and/or male physiology. Of notice, in our study, Se supplementation inhibited obesity-induced DNMT3A expression in epididymal adipose tissue. This could be related, at least in part, to the previously described anti-inflammatory actions by Se in our study. Although histone deregulation has been associated with obesity [52], we did not observe alterations in H3K4me3 and H4K16ac in epididymal adipose tissue after any treatment.

CYP19 is a key aromatase in estrogen biosynthesis that is responsible for converting androgens to estrogens and is associated with the inflammatory response [53]. We observed that obese animals presented increased expression of CYP19 in epididymal adipose tissue. Its high expression is associated with hormonal unbalance in the male body and damaged spermatogenesis [54]. Importantly, we observed that Se supplementation during obesity normalized CYP19 expression, suggesting that the micronutrient protection of male physiology occurred at the level of testosterone metabolism.

According to testicular morphological analysis, we observed in all group’s tubular structures with preserved architecture, accompanied by the formation of spermatids and spermatozoa within the normal limits. Similarly, Nematollahi et al. [55] observed that intervention with a high-fat diet did not alter the mice’s testicular morphological characteristics. Thus, we decided to evaluate potential alterations in sperm cells at the molecular level. MicroRNAs (miRNAs) have recently been shown to be important for spermatogenesis [56]. Obese animals presented altered epigenetic marks, including microRNA cells in spermatozoids [57]. We confirm these findings and show that obesity led to decreased levels of miR-15b in sperm cells and increased levels of miR-497. miR-15b is a member of the miR-15/16 family and is primarily expressed in testis and is vital for spermatogenesis. miR-497 increased expression has been reported in sperm and seminal plasma-derived extracellular microvesicles of men with spermatogenesis disturbances [58,59] and in plasma of men diagnosed with metabolic syndrome. Collectively our data on miRNA suggest that obesity’s deleterious effects on male physiology may have occurred at the epigenetic and metabolic levels during spermatogenesis. Of notice, Se supplementation during obesity normalized miR-15b and miR-497 levels, specifically in sperm cells. In addition, the micronutrient reduced levels of miR-200c, which is associated with metabolism and inflammation and, when down-regulated, is important to control male germ cell development [60]. To the best of our knowledge, this is the first study to show Se miRNAs modulatory effects on sperm cells during obesity and to suggest that Se protective effects on male physiology could involve reprogramming of epigenetic processes factors during spermatogenesis.

More recently, increased interest has been directed towards the impact of the future father’s health condition and nutrition status on his offspring’s health, as male gametogenesis is a highly plastic process and prone to environmental deregulation. McPherson et al. [18] showed in a seminal mice study that intervention with an antioxidant mix including Se mitigated not only obesity-induced miRNA deregulation in sperm cells but also improved fetal developmental parameters. Their data highlight the potential of supplementing future obese fathers with antioxidants, including Se, to improve their reproductive physiology and promote their offspring’s health. We previously showed that Se deficiency during the preconception program increased the risk of breast cancer in female offspring [61]. However, Se supplementation of lean animals in this same study did not exert protective effects [61]. Se supplementation efficacy depends on the individual metabolic context and oxidative stress context [62]. Thus, based on data from the present study showing that Se supplementation in a condition of metabolic and oxidative stress improved male physiology, it would be important to investigate in further studies if Se supplementation specifically during obesity would prevent breast cancer risk in daughters, as this paternal metabolic condition was also shown to program increased disease risk [57].

## 5. Conclusions

Altogether, our study advances the knowledge on the role of Se on obesity and reinforces its supplementation as a potential strategy to ameliorate this condition. In addition, to the best of our knowledge, this is the first study to show that Se supplementation during obesity mitigated obesity-induced deregulation of male physiology. This involved antioxidant and anti-inflammatory actions and adipogenesis and hormone-related pathways modulation in epididymal adipose tissue, as well as epigenetic reprogramming in sperm cells. Because these effects occurred during the transition between puberty and young adulthood, a developmental window where spermatogenesis is especially prone to environmental-induced disturbances, future clinical studies should focus on potential interventions with Se on this target population in order to improve male physiology. This could benefit not only themselves but also their future descendants.

## Data Availability

Data is available within the article.

## Supplementary Materials

The following are available online at https://www.mdpi.com/article/10.3390/antiox11050895/s1, Figure S1: Initial (a) and final (b) body weight and daily feed (c) and water (d) intake, Figure S2. Representative photomicrographs of epididymal adipose tissue sections stained with hematoxylin-eosin and marked with H3k4me3 antibody, Table S1: Control and high-fat diets composition, Table S2: Primer sequences used in real-time PCR for mRNA expression analysis, Table S3: Taqman assays used for the analysis of miRNA expression by qPCR (Thermo Fisher Scientific, Waltham, MA, USA).
