# Supplementary material for: Selenium Supplementation during Puberty and Young Adulthood Mitigates Obesity-Induced Metabolic, Cellular and Epigenetic Alterations in Male Rat Physiology
Source: Antioxidants (Basel). 2022 Apr 30;11(5):895. doi: 10.3390/antiox11050895 (PMC9138167; doi:10.3390/antiox11050895)
Supplement: Supplementary file 1 [file antioxidants-11-00895-s001.zip › antioxidants-1682960-supplementary.pdf]

Table S1. Control and high-fat diets composition.

	CO group		OB group	
	g/Kg	g/500 kcal	g/Kg	g/500 kcal
**Corn starch**	529.486	67.81	121.667	11.69
**Casein**	200	25.64	264	25.38
**Sucrose**	100	12.82	132	12.69
**Soy oil**	70	8.97	92	8.84
**Lard**	-	-	257	24.71
**Fiber**	50	6.41	66	6.34
**Mineral mix**	35	4.487	46	4.42
**Mix of vitamins**	10	1.282	13	1.25
**L-cystine**	3	0.38	4	0.38
**Choline bitartrate**	2.5	0.32	3.3	0.32
**Tert-Butylhydroquinone**	0.014	0.0018	0.037	0.0035
**Total**	1000	128.12	1000	96.02

Control diet (AIN-93G) containing 3.9 kcal/g and hyperlipidic lard-based diet containing 5.2 kcal/g. *For detailed mineral mix composition, please see Reeves et al. (1993) [19]. **The OBSe group received the high fat diet and supplementation with Se (0.45 ppm) as sodium selenate diluted in water.

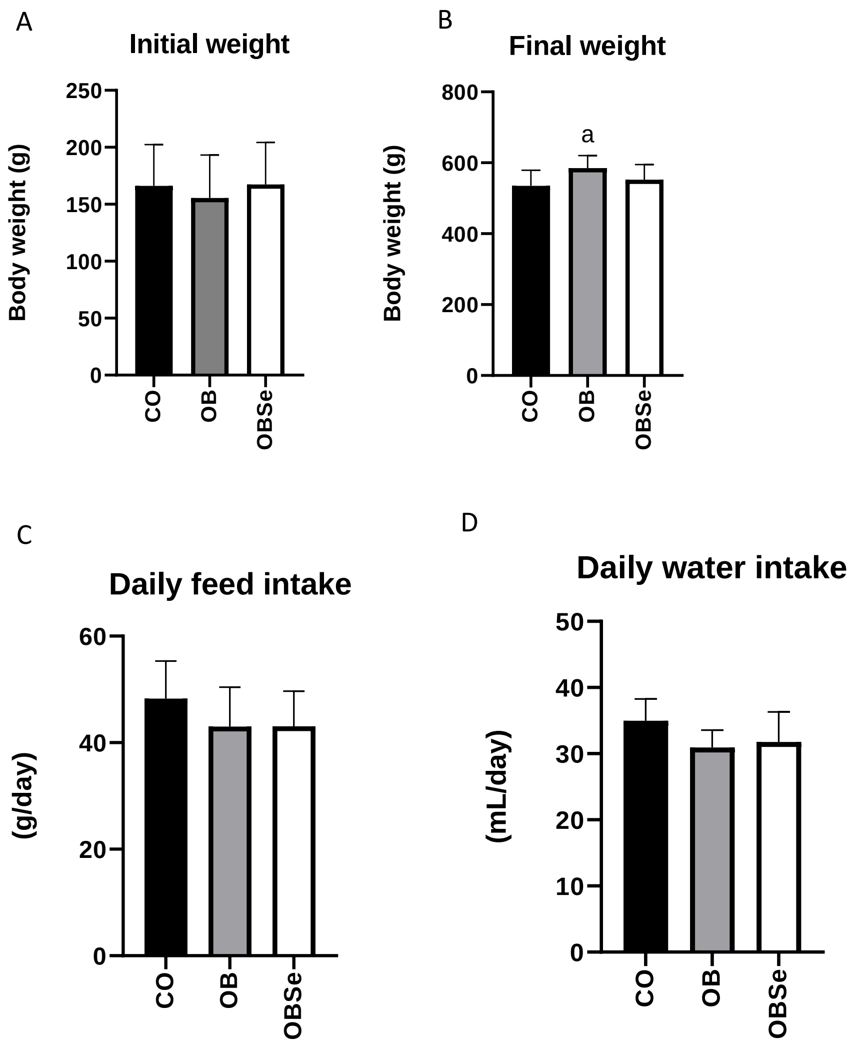

Figure S1. Initial (a) and final (b) body weight and daily feed (c) and water (d) intake. Data represent the mean ± SEM (n = 15/group). *Statistically significant difference ($p \leq 0.05$) compared to CO group according to one-way ANOVA, followed by Tukey's multiple comparisons test.

Table S2. Primer sequences used in real-time PCR for mRNA expression analysis.

Gene	Primer sequence (5′–3′)
B actin (Control)	F:GACAACGGCTCCGGCATGTGCAAAG<br>R: TTCACGGTTGGC CTTAGGGTTCAG
PPARγ	F: CTGGCCTCCCTGATGAATAAAG<br>R: AGGCTCCATAAAGTCACCAAAG
IL-6	F: GTGGAAGACAAACCATGTTGCCGT<br>R:TATTGCAGGTGAGCTGGACGTTCT
TNFα	F:AGAACAGCAACTCCAGAACACCCT<br>R:TGCCAGTTCCACATCTCGGATCAT
Adiponectin	F:GCGCTCCTGTTCCTCTTAAT<br>R:CATCCAACCTGCACAAGTTTC
C/EBPβ	F: GACTACGCAACACACGTGTAACT<br>R: CAAAACCAAAAACATCAACAACCC
C/EBPα	F: TTACAACAGGCCAGGTTTCC<br>R: GGCTG GCGACATACAGATCA
CYP1A1	F: CCATGACCAGGAACTATGGG<br>R: TCTGGTGAGCATCCAGGACA
DNMT3A	F: ACGCCAAAGAAGTGTCTGCT<br>R: CTTGGCTATTCTGCCGTGTT
DNMT1	F: GGTTCTGCGCGGGGACAGAC<br>R: CCGGCAACATGGCCTCAGGG

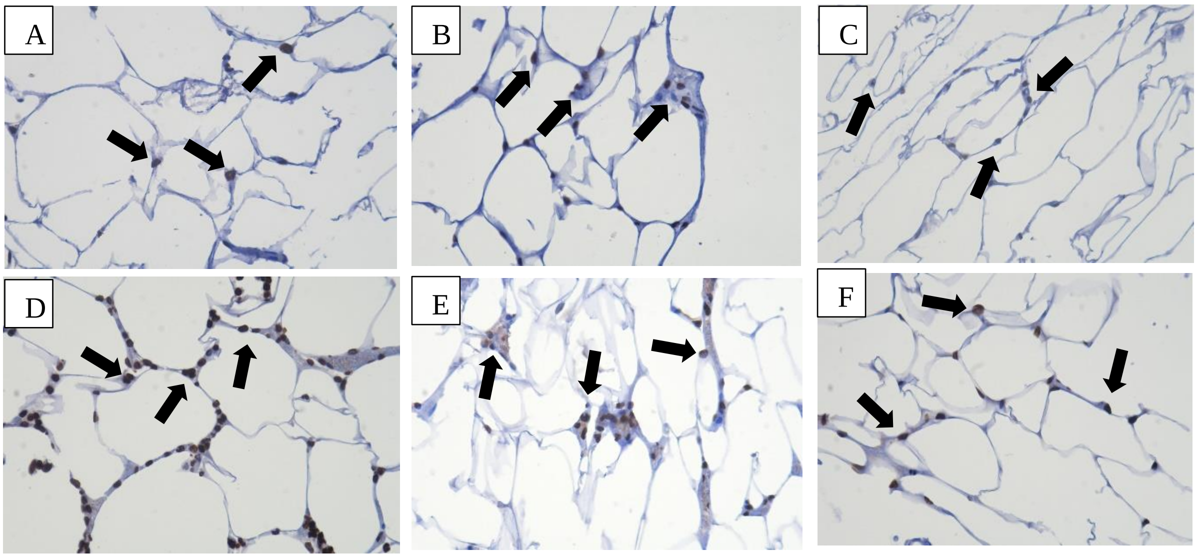

Figure S2. Representative photomicrographs of epididymal adipose tissue sections stained with hematoxylin-eosin and marked with H3k4me3 antibody [(A) CO group, (B) OB group, and (C) OBSe]; or with H4k16ac antibody [(D) CO group, (E) OB group, and (F) OBSe]. All images are at 10x. No statistically significant difference ($p \leq 0.05$) among groups according to one-way ANOVA, followed by Tukey's multiple comparisons test.

Table S3. Taqman assays used for the analysis of miRNA expression by qPCR (Thermo Fisher Scientific, USA)

**miRNA**	**Assay ID**
hsa-miR-200c-3p	002300
hsa-miR-497-3p	002368
Hsa-miR-15b	000390
RNU49	000209
